# Associations between frailty dimensions and quality of life in older adults with Type 2 diabetes Mellitus: A cross-sectional study

**DOI:** 10.1007/s40520-026-03428-x

**Published:** 2026-06-04

**Authors:** Dimitra Natsina, Fotini Malli, Maria Malliarou, Georgios Tsioumanis, Maria Saridi, Katerina Toska, Theodosios Paralikas

**Affiliations:** Department of Nursing, University of Thessaly, Gaiopolis Campus, Larissa- Trikala Ring-Road, Larissa, 415 00 Greece

**Keywords:** Type 2 diabetes mellitus, Frailty, Older adults, Geriatric assessment, Quality of life

## Abstract

**Introduction:**

Frailty is increasingly recognized as a clinically relevant condition in older adults with Type 2 Diabetes Mellitus (T2DM), with important implications for functional outcomes and individualized care. This study examined the association between multidimensional frailty and quality of life in older adults with T2DM.

**Methods:**

A cross-sectional study was conducted among 112 older adults aged ≥ 65 years in the Trikala Regional Unit, Central Greece. Frailty was assessed using the Tilburg Frailty Indicator (TFI), and quality of life was measured using the Older People’s Quality of Life Questionnaire (OPQOL-35). Overall quality of life was operationalized as the OPQOL-35 mean score. Pearson correlation analysis was performed to examine the association between quality of life and frailty components, and multiple linear regression analysis was used to examine independent associations with quality of life.

**Results:**

The sample demonstrated a moderate level of frailty (*M* = 7.88, *SD* = 2.78), exceeding the established cut-off for frailty. Physical fatigue and upper limb weakness were the most frequently reported physical limitations. All frailty components (physical, psychological, and social) were significantly associated with poorer OPQOL-35 mean scores (*p* < .01). The strongest correlation was observed for physical health-related problems (*r* = .690, *p* < .001), followed by social (*r* = .582, *p* < .001) and psychological components (*r* = .486, *p* < .001). The regression model explained 77.2% of the variance in quality of life (*R*² = 0.772), with physical frailty emerging as the strongest associated factor (β = 0.459, *p* < .001), followed by social (β = 0.303, *p* < .001) and psychological components (β = 0.202, *p* = .005).

**Conclusions:**

Frailty shows a strong multidimensional association with quality of life in older adults with T2DM. These findings highlight the importance of early frailty screening and support the implementation of individualized, multidisciplinary management strategies in primary care to preserve functional ability and overall well-being in this vulnerable population.

## Introduction

 Population ageing represents one of the most defining demographic and social transformations of the 21st century. Advances in medical science, public health, and socioeconomic development have led to a substantial increase in life expectancy worldwide, resulting in a rapid expansion of the population aged 60 and 65 years and older [1–3]. However, increased longevity is not necessarily accompanied by proportional improvements in functional health. Additional years of life are frequently characterized by chronic diseases, multimorbidity, and limitations in autonomy [4]. Within this context, contemporary scientific discourse has progressively shifted from the mere extension of survival toward the preservation of functional ability, independence, and quality of life in older adults [5], reflecting a central priority of geriatric medicine which is the prevention of disability and maintenance of functional independence.

Greece is among the most rapidly ageing countries in Europe, exhibiting a particularly high proportion of individuals aged 65 years and older, an increased median age, and an unfavorable old-age dependency ratio [6, 7]. Persistently low fertility rates, combined with the emigration of younger age groups and increasing life expectancy, have resulted in an increasingly inverted population pyramid. Concurrently, data on Healthy Life Years indicate that a substantial proportion of later life is experienced with functional limitations and chronic morbidity, imposing a considerable burden not only on health and social care systems but also on individual autonomy and well-being [6, 8, 9]. These demographic trends underscore the clinical importance of identifying modifiable factors that contribute to vulnerability in older adults.

Within this framework, type 2 diabetes mellitus (T2DM) emerges as a key disease of ageing, demonstrating particularly high prevalence in older age groups and close biological links to ageing processes [10–13]. Age-related alterations in pancreatic β-cell function, chronic low-grade inflammation, mitochondrial dysfunction, and cellular senescence constitute a pathophysiological substrate that promotes the development and progression of T2DM [14–17]. Consequently, diabetes is frequently conceptualized as a model of accelerated ageing, with important implications for functional capacity, resilience to stressors, and overall well-being in older individuals [16–18]. Beyond glycemic dysregulation, older adults with T2DM often present with multimorbidity, polypharmacy, increased risk of complications such as lower-extremity amputations, and greater susceptibility to adverse clinical outcomes [4, 19, 20].

The global prevalence of diabetes mellitus continues to rise, with a disproportionate burden observed among individuals aged 65 years and older [18]. In Greece, available epidemiological data indicate high rates of diabetes and prediabetes, marked geographical variation, and an increasing economic burden on the healthcare system [21–23]. However, beyond its economic and epidemiological dimensions, diabetes in later life is closely associated with the development of frailty, a geriatric syndrome characterized by reduced physiological reserve and increased vulnerability to internal and external stressors [24–26].

Frailty occurs more frequently among older adults with diabetes compared to their non-diabetic counterparts and is associated with increased risks of functional decline, hospitalization, treatment-related complications, and reduced quality of life [24, 25, 27]. Given its multidimensional nature, frailty requires reliable and clinically applicable assessment tools. The Tilburg Frailty Indicator (TFI) captures physical, psychological, and social domains of frailty through structured self-report measures, allowing a comprehensive evaluation of vulnerability beyond purely biomedical parameters [28]. This multidimensional approach is particularly relevant in geriatric diabetes care, as psychological distress and social isolation may amplify physical vulnerability, thereby influencing treatment outcomes and daily functioning [4]. In parallel, quality of life constitutes a fundamental outcome in geriatric assessment, reflecting the cumulative impact of chronic disease burden, frailty, and psychosocial factors on autonomy and overall well-being [27, 29].

Despite well-documented international evidence linking frailty and quality of life in older populations, evidence specifically addressing the multidimensional contribution of frailty domains to quality of life in older adults with T2DM remains limited, while the Greek literature in this field is particularly scarce [21, 22]. Moreover, most existing studies predominantly emphasize the physical dimension of frailty, often overlooking its psychological and social components and their independent as well as combined influence on quality of life [8, 30, 31]. Addressing this gap is clinically relevant, as comprehensive frailty assessment may inform individualized management strategies and multidisciplinary interventions in older adults with diabetes.

Therefore, the present study aimed to investigate the association between multidimensional frailty and quality of life among older adults diagnosed with T2DM and explore potential implications for frailty screening and individualized management in geriatric diabetes care.

## Methodology

### Study design and participants

A cross-sectional study design was employed. A convenience sample of 112 older adults aged ≥ 65 years with a confirmed diagnosis of Type 2 Diabetes Mellitus (T2DM) was recruited from hospitals, outpatient clinics and care facilities in Trikala Region Unit, Central Greece reflecting a mixed residential sample including individuals living independently, with family members, and a small proportion residing in nursing homes.

Eligibility criteria included: (a) age ≥ 65 years, (b) confirmed diagnosis of T2DM, (c) fluency in the Greek language, and (d) sufficient cognitive capacity to complete the questionnaires independently. Individuals with severe cognitive impairment or acute medical conditions at the time of assessment were excluded to ensure data validity.

The study was conducted in accordance with the principles of the Declaration of Helsinki. Ethical approval was obtained from the Internal Ethics and Deontology Committee of the Department of Nursing, University of Thessaly (Protocol No. 94/25-02-2026). Written informed consent was obtained from all participants prior to data collection.

### Study objectives

The primary objective was to investigate the association between multidimensional frailty and quality of life in older adults with T2DM. Specifically, the study aimed to assess frailty levels, evaluate quality of life, and examine the independent contribution of physical, psychological, and social frailty components to health-related quality of life. The study further sought to underscore the clinical importance of early frailty detection within Primary Health Care settings to inform individualized and multidisciplinary management strategies [30].

### Data collection and instruments

Data were collected between November 2025 and December 2025, using an anonymous structured questionnaire comprising demographic variables, the Tilburg Frailty Indicator (TFI), and the Older People’s Quality of Life Questionnaire (OPQOL-35).

The TFI is a 15-item self-reported multidimensional instrument assessing physical, psychological, and social domains of frailty. Total scores range from 0 to 15, with higher scores indicating greater frailty severity. A score ≥ 5 is commonly used to indicate frailty [28].

The OPQOL-35 consists of 35 items across eight domains of quality of life, along with a global item (“Life overall”) assessing overall life evaluation. Responses were rated on a five-point Likert scale (1 = Strongly Agree to 5 = Strongly Disagree). For the purposes of the present analysis, a composite quality of life score was computed as the mean of all OPQOL-35 items. All negatively worded items were reverse-coded so that higher scores consistently indicated poorer quality of life [32, 33].

### Statistical analysis

Data were analyzed using IBM SPSS Statistics (version 26). Internal consistency reliability was evaluated using Cronbach’s alpha (*α*), yielding acceptable reliability for the TFI (*α* = 0.69) and excellent reliability for the OPQOL-35 (*α* = 0.96). The combined scale also demonstrated very high reliability (*α* = 0.92).

Descriptive statistics were calculated for demographic characteristics and scale scores. Pearson’s correlation coefficients (two-tailed) were used to examine the associations between quality of life (OPQOL-35 mean score) and frailty components (physical, psychological and social). Pairwise deletion was applied to handle item-level missing data, resulting in minor variations in sample size across analyses. Multiple linear regression analysis was conducted with overall quality of life (OPQOL-35 mean score) as the dependent variable and physical, psychological, and social frailty components as independent variables. Assumptions of normality, linearity, and multicollinearity were examined prior to model estimation.

The sample size of 112 participants was deemed sufficient for the multiple linear regression analysis, as it exceeds the commonly recommended minimum of 10–15 observations per independent variables (3 variables), ensuring adequate statistical power to detect meaningful effects. Statistical significance was set at *p* < .05.

## Results

### Demographic characteristics of the sample

The study sample consisted of 112 older patients with Type 2 Diabetes Mellitus. The majority of participants belonged to the 71–80 and 65–70 age groups, while approximately one-fifth of the sample was aged over 80 years. Women outnumbered men, accounting for nearly two-thirds of the total sample.

Regarding marital status, the majority of participants were married, while about one-third were widowed. In terms of educational attainment, more than half of the participants had completed primary education or less, a finding that reflects the educational profile of older cohorts.

Most participants resided in urban areas and lived with their spouse, while approximately one-quarter lived alone. Regarding therapeutic management for Type 2 Diabetes, nearly half were receiving exclusively oral antidiabetic medication, while a significant proportion followed a combination therapy of insulin and tablets. The detailed demographic and clinical characteristics of the sample are presented in Table 1.

**Table 1:** Demographic Characteristics

Variable	Category	*n* (%)
Age (years)	65–70	35 (31.3)
	71–80	53 (47.3)
	81–90	21 (18.8)
	> 90	3 (2.7)
Gender	Female	64 (57.7)
	Male	47 (42.3)
Marital Status	Married	67 (60.4)
	Widowed	32 (28.8)
	Single	6 (5.4)
	Divorced	6 (5.4)
Education	Primary	47 (42.0)
	Secondary	12 (10.7)
	Tertiary	27 (24.1)
	Some primary school	17 (15.2)
	No formal education	9 (8.0)
Place of Residence	City	72 (69.9)
	Town	18 (17.5)
	Village	13 (12.6)
Living Arrangement	With spouse	61 (55.5)
	Alone	28 (25.5)
	With children	15 (13.6)
	Nursing home	6 (5.5)
T2DM Treatment	Tablets	54 (48.6)
	Insulin	16 (14.4)
	Combination	41 (36.9)

*n* = 112. Percentages are based on valid responses; missing data were excluded from percentage calculations.

### Frailty of older adults (Tilburg frailty indicator)

The frequency distributions of the 15 items of the Tilburg Frailty Indicator (TFI) are presented. The majority of participants reported good perceived physical health (73.2%) and an absence of recent unintentional weight loss (64.0%). Most did not report difficulties in walking (55.4%) or maintaining balance (76.6%), nor did they report vision (91.8%) or hearing (78.2%) problems.

However, a significant proportion of the sample reported physical fatigue (65.2%) and upper limb weakness (50.0%), elements suggesting the presence of physical limitations in a segment of the population.

At the psychological level, most participants stated that they experienced memory problems “sometimes” (43.1%), while symptoms of low mood (51.8%) and anxiety or nervousness (47.3%) were also reported primarily on an occasional basis. Nevertheless, the majority stated that they cope well with their problems (71.3%).

At the social level, most participants did not live alone (73.6%) and reported adequate social support (89.2%). A sense of lack of social companionship was reported mainly “sometimes” (43.1%) or not at all (42.2%).

Overall, the mean Tilburg Frailty Indicator (TFI) score was 7.88 (*SD* = 2.78), indicating a moderate to high level of frailty in the study population. Based on the established cut-off point (TFI ≥ 5), 91 participants (81.3%) were classified as frail. These findings are consistent with the observed frequency of physical limitations within the sample.

Item-level missing data were present in several TFI items (ranging between 0 and 3 responses per item). Analyses were performed using available-case (pairwise deletion), resulting in minor variations in the number of valid responses across items.

### Quality of life of older adults

The quality of life of the participants was assessed using the OPQOL-35 questionnaire. Descriptive statistics (means and standard deviations) were calculated for the individual domains of the questionnaire in a sample of 112 individuals. Responses were recorded on a five-point Likert scale (1 = Strongly Agree to 5 = Strongly Disagree), with higher values indicating poorer perceived quality of life (OPQOL-35 mean score). Negatively worded items were reverse-coded prior to the calculation of domain scores to ensure conceptual consistency across items.

**Table 2:** OPQOL-35 domain scores (mean item scores)

Dimension	M	SD
Life overall	2.23	0.85
Life in general	2.74	0.91
Health	2.80	1.04
Social relationships	2.06	0.79
Independence, control over life, freedom	2.42	0.91
Home and neighborhood	2.12	0.79
Psychological & emotional well-being	2.15	0.92
Financial situation	3.07	0.86
Leisure time & activities	2.90	0.92

*M* = Mean, *SD* = Standard Deviation. The item “Life overall” represents a single global evaluation item and is reported separately from the eight OPQOL-35 domains. Higher scores indicate poorer perceived quality of life (OPQOL-35 mean score).

As presented in Table 2, variability was observed across quality of life domains. The single global item “Life overall” showed a mean score of 2.23 (SD = 0.85), indicating a generally satisfactory overall life evaluation among participants. Lower mean values were observed in the domains of social relationships (M = 2.06, SD = 0.79), psychological and emotional well-being (M = 2.15, SD = 0.92), and home and neighborhood (M = 2.12, SD = 0.79), indicating relatively more favorable perceived quality of life in these areas.

Conversely, higher mean values, indicating relatively poorer perceived quality of life, were observed for financial situation (*M* = 3.07, *SD* = 0.86) and leisure time and activities (*M* = 2.90, *SD* = 0.92), suggesting greater perceived limitations in these areas.

Overall, despite living with a chronic condition, older adults with T2DM in the sample appeared to maintain satisfactory levels of quality of life, particularly in social and psychological dimensions, while financial status and leisure opportunities emerged as comparatively weaker areas.

### Correlation analysis: frailty – quality of life

To examine the relationship between frailty and quality of life in older adults with Type 2 Diabetes Mellitus, a Pearson correlation analysis was performed between overall quality of life (OPQOL-35 mean score) and the individual components of frailty (physical/health, psychological, and social).

**Table 3:** Correlation between Quality of Life (OPQOL-35 mean score) and Frailty Components (Pearson r)

	Quality of Life Overall (OPQOL-35 mean score)	Health Problems	Psychological Components	Social Components
Quality of Life Overall (OPQOL-35 mean score)	1	0.690**	0.486**	0.582**
Sig. (2-tailed)		0.000	0.000	0.000
N	111	109	106	108
Health Problems	0.690**	1	0.442**	0.472**
Sig. (2-tailed)	0.000		0.000	0.000
N	109	109	106	108
Psychological Components	0.486**	0.442**	1	0.269**
Sig. (2-tailed)	0.000	0.000		0.005
N	106	106	106	106
Social Components	0.582**	0.472**	0.269**	1
Sig. (2-tailed)	0.000	0.000	0.005	
N	108	108	106	109

Higher scores in the quality of life scale indicate lower perceived quality of life, whereas higher scores in frailty components indicate greater frailty. ** Correlation is significant at *p* < .01 level (2-tailed).

As shown in Table 3, all components of frailty were positively and statistically significantly correlated with overall quality of life (OPQOL-35 mean score) (*p* < .01) indicating that higher levels of frailty are associated with poorer perceived quality of life. Specifically, the highest correlation is observed between quality of life and health problems (*r* = .690, *p* < .001). Moderate but statistically significant correlations are also observed with social (*r* = .582, *p* < .001) and psychological components (*r* = .486, *p* < .001). These findings demonstrate that beyond the physical dimension, both social and psychological factors contribute substantially to reduced quality of life in older adults with T2DM.

### Multiple linear regression

To further examine the association between frailty components and quality of life, a multiple linear regression analysis was performed with overall quality of life (OPQOL-35 mean score) as the dependent variable and the physical (health problems), psychological, and social components of frailty as independent variables. The results of the regression analysis are presented in Table 4.

**Table 4:** Multiple linear regression with Quality of Life as the dependent variable

	B	Std. Error	Beta	t	Sig.
(Constant)	1.574	0.070		22.397	< 0.001
Health Problems	0.866	0.147	0.459***	5.894	< 0.001
Psychological Components	0.202	0.070	0.202**	2.869	0.005
Social Components	0.399	0.095	0.303***	4.182	< 0.001

* *p* < .05, ** *p* < .01, *** *p* < .001. Dependent Variable: Quality of Life (OPQOL – 35 mean score). *R*² = 0.772. The regression model was based on *N* = 106 complete cases (listwise deletion).

The proposed regression model explains 77.2% of the variance in overall quality of life (OPQOL-35 mean score, higher values indicate poorer quality of life) (*R*^2^ = 0.772), indicating a substantial explanatory capacity within the present sample. All components of frailty were significantly associated with quality of life. Specifically, health problems showed the strongest association (β = 0.459, *p* < .001), followed by social (β = 0.303, *p* < .001) and psychological components (β = 0.202, *p* = .005). Given the coding of the quality of life scale, where higher scores indicate poorer perceived quality of life, these positive coefficients indicate that higher levels of frailty are associated with worse quality of life.

It should be noted that the model is unadjusted for sociodemographic and clinical covariates; therefore, the reported estimates reflect associations within a single multivariable model rather than confounder-adjusted independent effects. The regression analysis was conducted using listwise deletion due to item-level missing data, resulting in a final analytic sample of *N* = 106 participants with complete data across all variables included in the model.

The regression equation is as follows:

Quality of Life = 1.574 + 0.866(Health Problems) + 0.202(Psychological Components) + 0.399(Social Components).

Overall, the findings indicate a consistent association between frailty and quality of life in older adults with T2DM, highlighting the multidimensional nature of this relationship.

## Discussion

The present study investigated the relationship between frailty and quality of life among older adults with Type 2 Diabetes Mellitus in the Trikala Region Unit, Central Greece. Focusing on this specific population is particularly relevant, as aging is accompanied by an increased incidence of diabetes and a greater burden on functional status and healthcare requirements [11, 15, 30]. In Greece, the prevalence of diabetes increases significantly with age, necessitating the investigation of associations that influence the well-being of older adults beyond glycemic control [21, 22].

A key finding of this study was the statistically significant association between frailty and quality of life. Specifically, increased health problems, as well as the psychological and social components of frailty, were associated with lower levels of quality of life. Multiple linear regression analysis demonstrated that all three dimensions of frailty were significantly associated with quality of life, with the physical domain exhibiting the most substantial impact [26, 29, 30].

Based on the total TFI score, the sample demonstrated a moderate level of frailty, exceeding the commonly used cut-off point for frailty. This finding indicates that a substantial proportion of participants can be considered frail, despite relatively preserved psychological and social functioning. The higher burden observed in the physical domain, particularly fatigue and upper limb weakness, further supports the clinical relevance of physical vulnerability in this population. These findings are reinforced by recent research on older adults in Crete, where 14.8% of the population was characterized as frail and 34.1% as pre-frail, confirming that a significant proportion of older adults in the Greek provinces maintain a level of functionality but remain at risk of transitioning to a more severe state of frailty. That study also demonstrates a close association between good quality of life, functional independence and lower frailty levels, aligning with the observations of the present work [30]. Additionally, the results are consistent with the biopsychosocial model of the TFI, which approaches frailty as a multidimensional phenomenon rather than exclusively as physical decline [28]. Importantly, the use of the TFI in this context supports its applicability as a practical screening tool in clinical settings, enabling early identification of vulnerable older adults with T2DM. This has direct implications for comprehensive geriatric assessment and the implementation of multidisciplinary, patient-centered management strategies aimed at preventing functional decline and optimizing quality of life [18, 19, 27].

Regarding quality of life, participants reported a generally good perceived status, with better performance in the dimensions of health, psychological well-being, and social relationships, while dimensions related to financial situation and participation in leisure activities were less favorable. These results are consistent with previous studies indicating that social support and psychological well-being are critical determinants of quality of life in older adults, even in the presence of chronic diseases [34]. In this context, Abd Ghafar et al. [27] emphasize that the combination of diabetes and frailty acts synergistically, causing significant degradation in psychosocial well-being and overall quality of life (OPQOL-35 mean score).

Overall, these findings align with international literature documenting that frailty is related to diminished functional capacity, loss of autonomy, increased psychological burden, and limited social participation, elements that directly affect the quality of life of older adults [24–26]. Specifically, a study by Lin et al. [35] reported that among older adults with Type 2 diabetes, the prevalence of frailty reached 26.6%, while depression emerged as a significant factor associated with frailty. This finding further supports the multidimensional nature of frailty, extending beyond physical decline to include important psychological determinants. In older adults with diabetes, this relationship appears even more pronounced, as diabetes may exacerbate frailty through muscle mass loss, chronic inflammation, recurrent hyperglycemic episodes, and psychosocial burden [24, 27, 31].

The relationship between diabetes and frailty has been described as bidirectional; diabetes contributes to the development of frailty, while frailty complicates the management of the chronic disease and increases the risk of adverse outcomes [27]. Recent research in a sample of Greek older adults highlighted that the presence of chronic diseases, such as Type 2 diabetes, is directly linked to increased frailty levels, confirming the need for early intervention [30, 31]. The results of the present study reinforce the importance of systematic frailty assessment as an integral part of care for older adults with diabetes. To this end, contemporary guidelines and consensus statements emphasize that the recognition of frailty should lead to the individualization of therapeutic goals, with an emphasis on avoiding hypoglycemia and simplifying medication regimens to ensure patient safety and quality of life [18, 19].

## Conclusions

At the population level, the findings of this study acquire particular significance within the context of the rapid aging of the Greek population and the increasing burden of chronic conditions on healthcare systems. However, these findings also have direct clinical relevance, as the systematic integration of frailty assessment into Primary Health Care can facilitate early identification of vulnerable older adults with T2DM and support individualized, multidisciplinary management strategies. Such approaches may contribute to improved functional outcomes, optimized care planning, and the preservation of autonomy in this high-risk population. From this perspective, frailty emerges not only as a clinical concept but also as a meaningful indicator of public health planning and social policy in aging societies.

### Study limitations

The present study has several limitations. First, the cross-sectional design does not allow for causal inferences regarding the observed associations between frailty and quality of life. Second, the use of self-reported questionnaires may have introduced information bias due to subjective interpretation of items.

Third, the sample was recruited using convenience sampling from hospitals, outpatient clinics, and care facilities within a specific geographical region, which may have introduced selection bias and limits the generalizability of the findings. This sampling strategy may also have contributed to the relatively high prevalence of frailty observed in the study population (81.3%), potentially reflecting a more clinically complex subgroup.

Finally, important clinical information related to diabetes severity and management, including disease duration, glycaemic control (e.g., HbA1c), presence of complications, comorbidity burden, and medication load, was not available. These factors may act as relevant confounders in the relationship between frailty and quality of life and could not be controlled for in the analyses [36].

### Directions for future research

Future research should also incorporate more comprehensive clinical and metabolic data in individuals with Type 2 Diabetes Mellitus, including disease duration, glycaemic control (e.g., HbA1c), comorbidity burden, and medication use, in order to allow better adjustment for potential confounding factors in the relationship between frailty and quality of life. In addition, future studies could apply multivariable models with full covariate adjustment and sensitivity analyses to strengthen the robustness of observed associations.

## Data Availability

The datasets generated and analyzed during the current study are not publicly available due to privacy restrictions but are available from the corresponding author on reasonable request.
